# Isolation and structure elucidation of phenazine derivative from *Streptomyces* sp. strain UICC B-92 isolated from *Neesia altissima* (Malvaceae)

**Published:** 2020-04

**Authors:** Rina Hidayati Pratiwi, Iman Hidayat, Muhammad Hanafi, Wibowo Mangunwardoyo

**Affiliations:** 1 Department of Mathematics and Natural Sciences, Faculty of Post Graduated, Universitas Indraprasta PGRI, South Jakarta, Indonesia; 2 Department of Microbiology, Research Center for Biology, Indonesian Institute of Sciences (LIPI), Cibinong, Indonesia; 3 Research Center for Chemistry, Indonesian Institute of Sciences (LIPI), PUSPIPTEK, Serpong, Indonesia; 4 Department of Biology, Faculty of Mathematics and Natural Sciences, Universitas Indonesia, Depok, Indonesia

**Keywords:** Actinomycetes, Antibacterial, Endophyte, Gram-positive bacteria

## Abstract

**Background and Objectives:**

Endophytic actinomycetes have been known as a promising source for new antibiotics discovery against susceptible and resistant forms of pathogenic microorganisms. This study was aimed at determining antibacterial compound from *Streptomyces* sp. strain B-92 isolated from a medicinal plant *Neesia altissima*.

**Materials and Methods:**

*Streptomyces* sp. strain UICC B-92 was endophytic actinomycetes of *N. altissima* that obtained from Universitas Indonesia Culture Collection (UICC). Isolation and determination of bioactive compound were carried out using thin layer chromatography (TLC), nuclear magnetic resonance spectroscopy (NMR), and liquid chromatography mass spectrometry (LC-MS) analyses. An *in vitro* antibacterial assay of pure bioactive compound from the endophytic actinomycetes strain was performed against *Bacillus cereus* strain ATCC 10876, *Escherichia coli* strain ATCC 25922, *Salmonella typhimurium* strain ATCC 25241, *Shigella flexneri* strain ATCC 12022 and *Staphylococcus aureus* strain ATCC 25923.

**Results:**

The bioactive compound was identified as 4-((3S,4R,5S)-3,4,5-trihydroxy-6-(hydroxymethyl) tetrahydro-2H-pyran-2-yloxy) phenazine-1-carboxylic acid. *In vitro* antimicrobial assay showed that bioactive compound of *Streptomyces* sp. strain UICC B-92 exhibited antagonistic activities against two Gram-positive bacteria, viz, *B. cereus* strain ATCC 10876 and *S. aureus* strain ATCC 25923.

**Conclusion:**

The findings of this research showed that, bioactive compound of *Streptomyces* sp. strain UICC B-92 is suggested a new compound based on glycoside structure and its position.

## INTRODUCTION

Accelerating number of bacterial and fungal infections worldwide has become problematic disease due to antibiotic resistance of those pathogenic microorganisms (1). The problem is worsened by the emergence of new pathogens with the potential for rapid global spread, such as *Staphylococcus* spp., *Mycobacterium tuberculosis* and *Streptococcus* spp. (2). There are more interests in discovery and development of new antimicrobial agents from various sources to combat microbial resistance, including microbial endophytes associated with various medicinal plants (3).

Each of plant species generally hosts to one or more microbial endophyte species. Majority of microbial endophytes produce bioactive compounds that inhibit the growth of other microbes, and even acquire the ability to synthesize the same bioactive compounds produced by their host plant (3). Therefore, microbial endophytes are potential source for discovery of novel bioactive compounds for use in medicine, agriculture, and other industries. Among various microbial groups, members of actinomycetes have been known for their pharmaceutically important compounds as they have contributed 52.73% of antibiotic production of which approximately 50% for human consumption (4). These include munumbicins produced by *Streptomyces* sp. strain NRRL 30562 (5), kakadumycins produced by *Streptomyces* sp. strain NRRL 30566 (6), bioactive compound of multicyclic indolosesquiterpenes, i.e. Xiamycin B [1b], Xiamycin A [1a], Indosespene [2], and Sespenine [3] isolated from *Streptomyces* sp. strain HKI 0595 (7), coronamycin produced by *Streptomyces* sp. strain MSU-2110 (8) and so on.

*Neesia altissima* (Blume) Blume (Indonesian: Bengang) is a tropical plant belong to family Malvaceae. This plant has different local names in several regions in Indonesia such as ki bengang (Sundanese), and Durian hantu or sibengang (Sumatera) (9). The plant is a large tree (grows up to + 40 m) and distributed primarily in the rainforest of Malaysia and Indonesia (Sumatera, Borneo, and Java islands). This plant is endemic to Indonesia and used as treatment of gonorrhea, diuretic, and diarrhea diseases (10). Our previous study on discovery of new compound from microbial endophytes associated with *N. altissima* reported that three endophytic bacterial strains, viz, *Pseudomonas aeruginosa* strain UICC B-40 and strain UICC B-93, and *Pseudomonas azotoformans* strain UICC B-91, exhibited antibacterial activities against *Bacillus cereus* ATCC 10876, *Escherichia coli* ATCC 25922, *Salmonella* Typhimurium ATCC 25241, *Shigella flexneri* ATCC 12022, and *Staphylococcus aureus* ATCC 25923 (11). The (2E,5E)-phenyltetradeca-2,5-dienoate (MW = 300 g/mol) bioactive compound was isolated and determined from fermentation extract of *P. aeruginosa* strain UICC B-40, a bacterial endophyte strain from *N. altissima* (12).

The naturally occurring phenazines and many of its derivatives are found in nature produced by Gram-positive bacteria, particularly members of *Streptomyces* spp. (13). These include *S. griseoluteus* (14), *S. kebangsaanensis* (13), *S. lomondensis* (15), *S. anulatus* (16), *S. niveus* (17) and several other species (14, 18, 19). Luo et al. (14) also noted that members of *Streptomyces* have commonly been used as a source of various phenazines complex such as griseolutein, phenacetin, lomofungin, and 5,10-dihydrophenazine. In this study, we determined and elucidated the phenazine derivative antibacterial compound that isolated from endophytic actinomycetes from *N. altissima*. A mechanism of the antibacterial compound is morphologically described using Scanning Electron Microscope (SEM).

## MATERIALS AND METHODS

### Microbial sources.

*Streptomyces* sp. strain UICC B-92 was obtained from Universitas Indonesia Culture Collection (UICC) (20). This endophytic actinomycetes strain was isolated from *N. altissima* using the modification method published by Cao et al. (21).

### Isolation of bioactive compound.

Isolation of antibacterial compound from actinomycetes was conducted according to the method described by Pratiwi et al. (12) and Saha et al. (22) as follows: selected endophytic actinomycetes strain that showed highest antagonistic activity was sub-cultured on ISP2 medium and incubated for 7 days. Spores of the endophytic actinomycetes strain were inoculated into 500 mL of ISP2 broth medium (pH 6.75) in a 1 L Erlenmeyer flask and incubated in shaking incubator (100 rpm) at room temperature for 5 days. Fermented liquid was further extracted by ethyl acetate. The mixture was allowed to form two distinct layers and then filtrated. After filtration, 50 mL of fermented broth was taken in a 250 mL separating funnel. About 25 mL of different organic solvents (nonpolar to polar) were used to recover different products. The mixture was shaken for 15 min, and was kept in stationary condition for another 15 min to separate the solvent (ethyl acetate) from aqueous phase. The solvent was evaporated in a rotary evaporator. The obtained powder of crude extract was further dissolved in 2–5 mL methanol for thin layer chromatography (TLC) assay. The TLC plates were developed with CH _2_ Cl _2_ /10% MeOH solvent system. The plates were dried under hot air and were further stained with Anisaldehyde/ H _2_ SO _4_ and Ehrlich’s reagents, separately. Visualization was conducted under UV light (at λ254 nm and λ366 nm) for the appearance of different color bands. The components showing UV absorbance and fluorescence were marked and scanned.

### Identification of bioactive compound.

After 5 days incubation of the culture, the fermentation was stopped. Cells of actinomycetes were removed from fermentation broth by filtration and the culture filtrate was extracted by ethyl acetate. Purification of crude bioactive compound was conducted according to the method described by Pratiwi et al. (23) in the silica gel chromatography column (22 × 5 cm, Silica gel 60, Merck) and eluted with gradient solvent system consisting of ethyl acetate + hexane. Elutions collected during column chromatography were concentrated and tested for their antimicrobial activity against *B. cereus* and *S. aureus* to screen bioactive fractions. Further purification of bioactive fractions was carried out in HPLC preparative column at 3 mL/min flow rate. Structural elucidation of pure bio-active compounds from the strain was carried out by LC-MS and ^1^ H-NMR spectral studies (24).

### Antibacterial activity assay of pure bioactive compound.

An *in vitro* antibacterial assay of pure bioactive compound from the endophytic actinomycetes strain was performed against *B. cereus* strain ATCC 10876, *E. coli* strain ATCC 25922, *S.* Typhimurium strain ATCC 25241, *S. flexneri* strain ATCC 12022 and *S. aureus* strain ATCC 25923. All bacterial isolates used in this study are preserved at Universitas Indonesia Culture Collection (UICC).

The antibacterial activity assay was conducted using the disc diffusion method described by Pratiwi et al. (12) and Schwalbe et al. (25). Pathogenic bacterial isolates were inoculated onto 50 mL NB medium (Merck) in a 250 mL Erlenmeyer flask and were incubated at 37 ºC for 18 h. 200 μL of 10 ^5^ CFU/mL of each pathogenic bacterial cultures was applied to the surface of the NA plate (15 cm diam.). The bioactive compounds were prepared at concentrations of 100; 1,000; 5,000; 10,000; 50,000; and 250,000 μg/mL in 1% DMSO. Tetracycline (100 μg/mL) was used as positive control, and DMSO without bioactive compound was used as negative control. Approximately 15 μL of each concentration was diffused onto a 6 mm diameter disc (Fuoroni), and then placed on NA plates. Antimicrobial activity was observed after 24–48 h incubation at 37 °C. Inhibition activity was determined by measuring the diameter of the inhibition zone.

### Analysis of cellular morphology using scanning electron microscopy (SEM).

SEM was carried out according to the method described by Kai et al. (26). After treatment with antibacterial compound, 50 mL of bacterial cells with concentration of 10 ^8^ CFU/mL were soaked in glutaraldehyde 2.5% for 2 h, then washed with cacodylate buffer, and soaked in osmium tetraoxide solution for 4 h. The cells were further washed with cacodylate buffer and soaked in tanin acid solution for 12 h. The cells were hydrated with 5–10 mL EtOH 50%, 70%, 80%, 90% and 99%. Centrifugation was conducted at 3,500 rpm for 10 min. Cells were dissolved in tert-BuOH and coated with gold using a vacuum (6–7 Pa) for 20 min. Appearance of each damaged cells was carried out with the SEM system (JOEL seri JSM-5310LV) at 5,000× magnification.

## RESULTS

### Isolation of bioactive compound.

Approximately 27 fractions were determined in TLC analysis of the extract from actinomycetes strain B-92. Among them, fractions 9–13 showed highest antibacterial activity based on bioautography analysis (data not shown). Retention factor (Rf) value of these fractions were 0.56–0.58 mm, indicating that the fractions belong to polar compound. These fractions were further analyzed using spectroscopy method.

### Identification of bioactive compound.

**Fig. 1.**
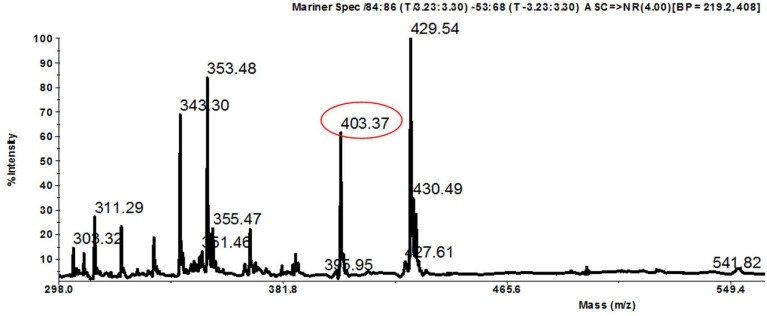
Spectral LC-MS of bioactive compound from endophytic *Streptomyces* sp. strain UICC B-92

**Fig. 2.**
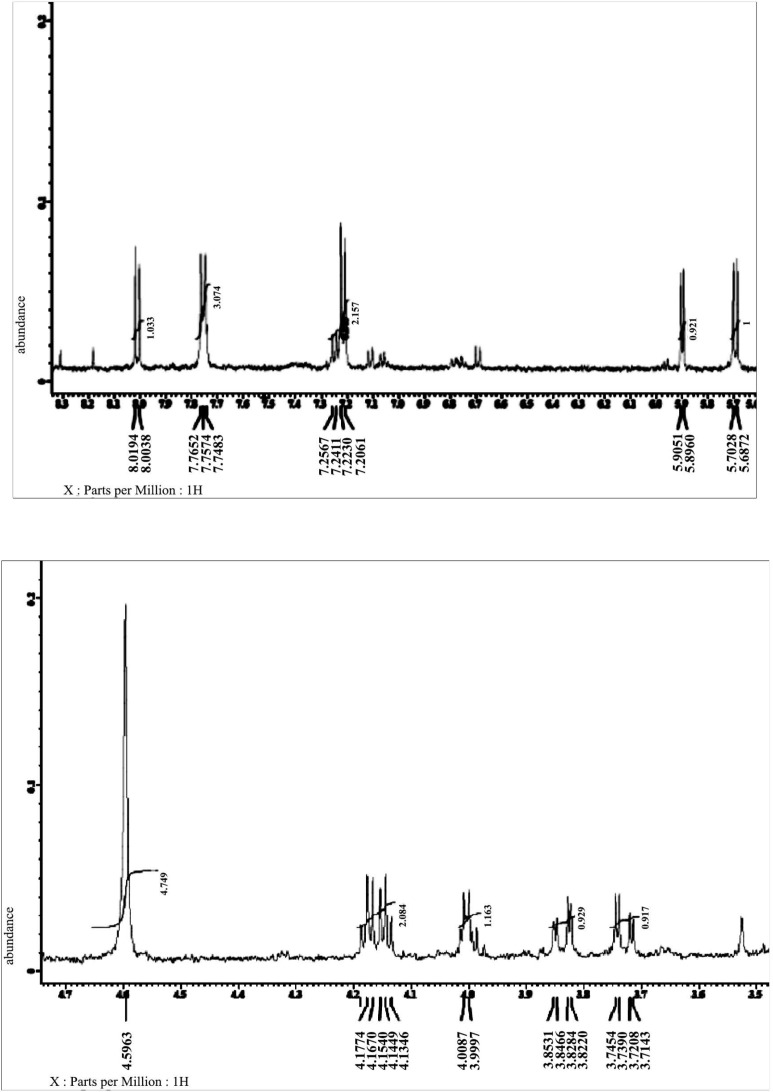
Spectral ^1^ H-NMR of bioactive compound from *Streptomyces* sp. strain UICC B-92

**Fig. 3.**
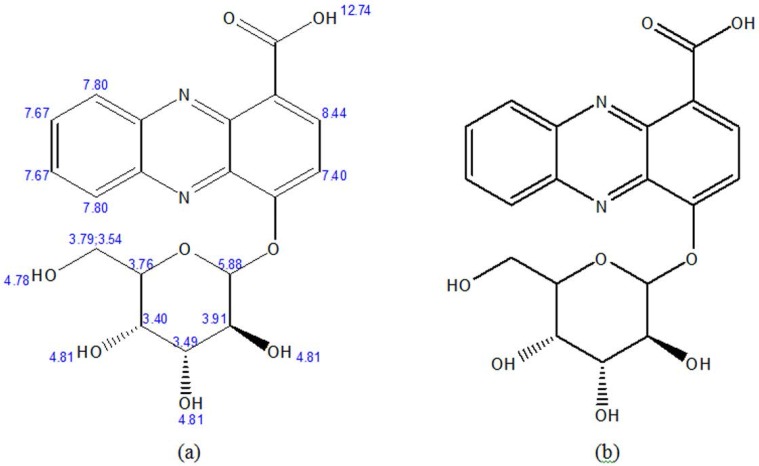
Aromatic signal of the purified compound from *Streptomyces* sp. strain B-92 at δH 5.7 (H; d; 8.3 Hz) ppm

**Fig. 4.**
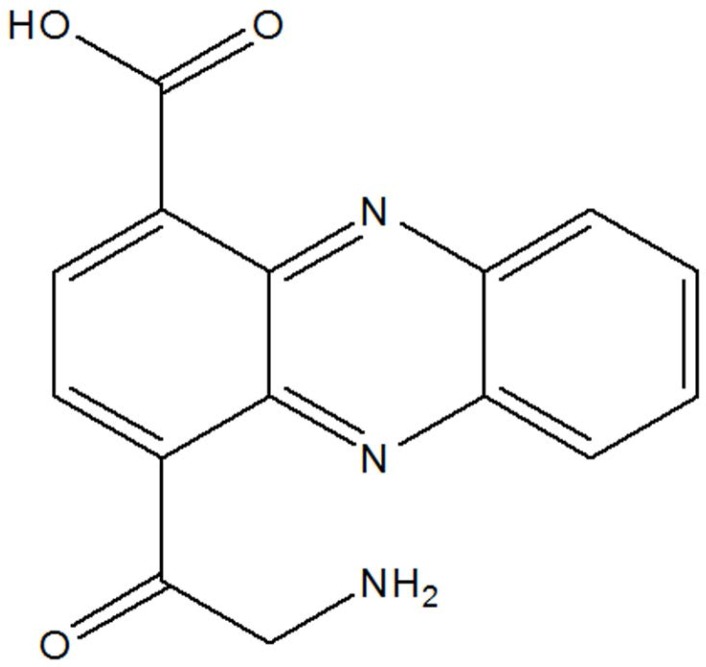
Phenazine structure of the purified compound from *Streptomyces* sp. strain B-92 at δH 7.75 (2H; d; 8.5 Hz); 7.22 (2H; d; 8.5 Hz) ppm

**Fig. 5.**
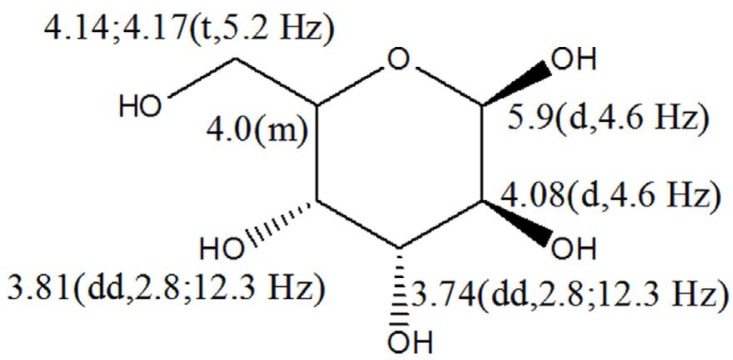
Sugar of the purified compound from *Streptomyces* sp. strain B-92 at δH 5.9 (H; d; 4.6 Hz); 4.08 (H; d; 4.6 Hz); 3.74 (H; dd; 2.8; 12.3 Hz); 3.81 (H; dd; 2.8; 12.3 Hz); 4.0 (m); 4.14; 4.17 (H; t; 5.2 Hz) ppm

**Fig. 6.**
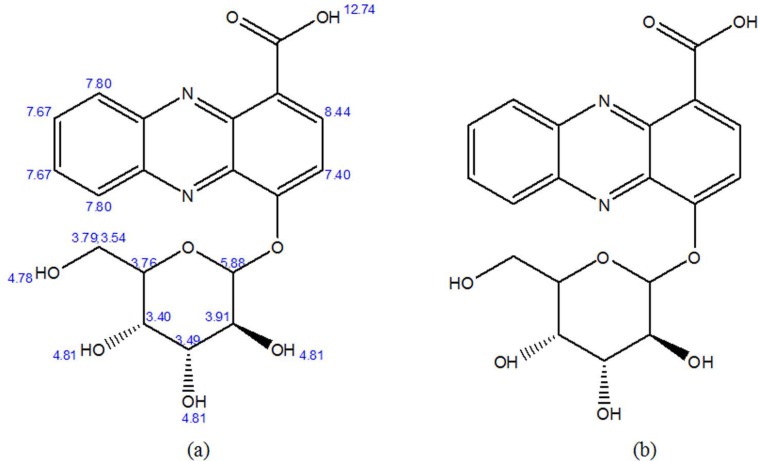
The structure of 4-O-glucosyl, 1-carboxyl-phenazine of the purified compound from *Streptomyces* sp. strain B-92: Prediction (a); Based on result (b)

LC-MS analyses (Fig. 1) and ^1^ H-NMR spectra (Fig. 2) of the purified compound from actinomycetes strain B-92 showed that fractions 9–13 exhibited aromatic signal at δH 5.7 (H; d; 8.3 Hz) ppm (Fig. 3), phenazine structure at δH 7.75 (2H; d; 8.5 Hz); 7.22 (2H; d; 8.5 Hz) ppm (Fig. 4), and sugar at δH 5.9 (H; d; 4.6 Hz); 4.08 (H; d; 4.6 Hz); 3.74 (H; dd; 2.8; 12.3 Hz); 3.81 (H; dd; 2.8; 12.3 Hz); 4.0 (m); 4.14; 4.17 (H; t; 5.2 Hz) ppm (Fig. 5). Therefore, based on these analyses, the fractions from purified compound of actinomycetes strain B-92 was determined as 4-((3S,4R,5S)-3,4,5-trihydroxy-6-(hydroxymethyl) tetrahydro-2H-pyran-2-yloxy) phena zine-1-carboxylic acid or 4-O-glucosyl, 1-carboxyl-phenazine with chemical formula C _19_ H _18_ N _2_ O _8_ (MW 402 g/mol) (Fig. 6 and Table 1).

**Table 1.** Spectral 1H-NMR of the bioactive compound 4-((3S,4R,5S)-3,4,5-trihydroxy-6-(hydroxymethyl) tetrahydro-2H-pyran-2-yloxy) phenazine-1-carboxylic acid (C _19_ H _18_ N _2_ O _8 _) that have 402 of molecular weight

**No.**	**1(CD_3_ OD, 500 MHz)**	**No.**	**1 (CD_3_OD, 500 MHz)**
	**δ^1^H(m, JHz) (ppm)**		**δ^1^(m, JHz) (ppm)**
	**Bioactive compound of *Streptomyces* sp. strain UICC B-92**		**Prediction of bioactive compound (using Chem Draw)**
1	-	1	-
2	8.01 (d; 8.3)	2	7.40 (d)
3	5.7 (d; 8.3)	3	8.44 (d)
4	-	4	-
5/8	7.75 (2H; d; 8.5)	5/8	7.80 (d)
6/7	7.22 (2H; d; 8.5)	6/7	7.67 (d)
1a	-	1a	-
4a	-	4a	-
5a	-	5a	-
8a	-	8a	-
1″	5.9 (d; 4.6)	1″	5.88 (d)
2″	4.08 (d; 4.6)	2″	3.91 (d)
3″	3.74 (dd; 2.8; 12.3)	3″	3.49 (dd)
4″	3.81 (dd; 2.8; 12.3)	4″	3.40 (dd)
5″	4.0 (m)	5″	3.76 (m)
6″	4.14; 4.17 (t; 5.2)	6″	3.79; 3.54 (t)

### Antibacterial activity assay.

**Fig. 7.**
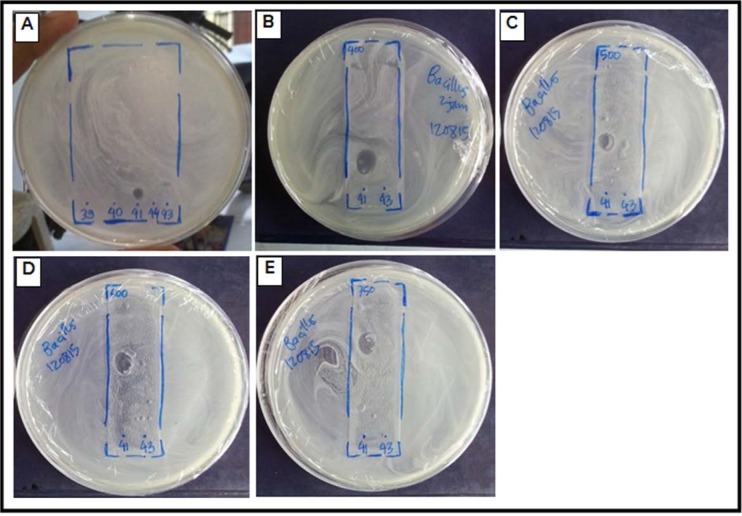
The bioautography visualization of the antibacterial bioactive compound inhibition zone from *Streptomyce*s sp. strain UICC B-92 for *B. cereus* at 41^st^ fraction with Methanol: Chloroform eluent = 2000 μL:250 μL (A); Methanol: Chloroform eluent = 2000 μL:400 μL (B); Methanol: Chloroform eluent = 2000 μL:500 μL (C); Methanol: Chloroform eluent = 2000 μL:600 μL (D); Methanol: Chloroform eluent = 2000 μL:750 μL (E)

Disc diffusion method of the purified compound isolated from the actinomycetes strain UICC B-92 exhibited antibacterial activity against *B. cereus* ATCC 10876, *S. aureus* ATCC 25923, and *S. flexneri* ATCC 12022. However, negative results for the antibacterial test were found on *E. coli* strain ATCC 25922 and *S.* Typhimurium strain ATCC 25241. Based on the inhibition zone diameter, *B. cereus* ATCC 10876 were the most sensitive bacterium (Fig. 7), followed by *S. aureus* ATCC 25923 and *S. flexneri* ATCC 12022 (Table 2).

**Table 2.** Antibacterial activity of the purified compound from *Streptomyces* sp. strain UICC B-92

**Compound concentration (μg/mL)**	**Diameter of inhibitory zone (mm)**				

	***B. cereus* strain ATCC 10876**	***E. coli* strain ATCC 25922**	***S. typhimurium* strain ATCC 25241**	***S. flexneri* strain ATCC 12022**	***S. aureus* strain ATCC 25923**
250,000	10.00	-	-	8.00	6.70
50,000	7.10	-	-	-	-
10,000	7.00	-	-	-	-
5,000	-	-	-	-	-
1,000	-	-	-	-	-
100	-	-	-	-	-
Tetracycline (1,000)	9.00	9.00	9.00	9.00	9.00
DMSO	-	-	-	-	-

(−): without inhibition halo

### The mechanism of action of the antibacterial compound.

**Fig. 8.**
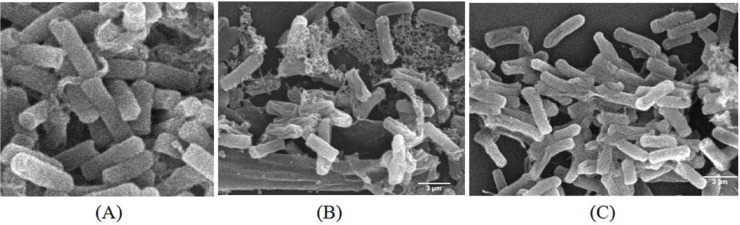
SEM analysis of *B. cereus* strain ATCC 10876 before and after antibacterial compound treatment from *Streptomyces* sp. strain UICC B-92. Negative control (1% DMSO), cells of *B. cereus* strain ATCC 10876 (A); cells of *B. cereus* strain ATCC 10876 bacterial cells after treatment with 250,000 μg/mL of the antibacterial compound from *Streptomyces* sp. strain UICC B-92 (B, C)

The SEM analysis of pathogenic bacterial cells indicated a possible mechanism of action of the antibacterial compound produced by *Streptomyces* sp. strain UICC B-92 (Fig. 8). Selection of *B. cereus* ATCC 10876 in the SEM analysis due to lowest concentration (the minimal inhibitory concentration / MIC) of the bioactive compound from *Streptomyces* sp. strain UICC B-92 was found against this bacterium (10,000 μg/mL) (Table 2). The SEM analysis revealed that bacterial cells of *B. cereus* strain ATCC 10876 morphologically changed after treatment using the bioactive compound of *Streptomyces* sp. strain UICC B-92. A surface of the *B. cereus* strain ATCC 10876 cells in the control group were smooth and the bacteria cells were plump and cylindrical. However, the cell membranes became coarse, wrinkled, and distorted (Fig. 8B and C) after treatment with the antibacterial compound from *Streptomyces* sp. strain UICC B-92. Local rupture and pore formation were also observed in the cell membranes (Fig. 8B and C).

## DISCUSSION

*N. altissima* has been known by the local people in Southeast Asian, in particular Indonesia, as a plant to treat infectious diseases such as gonorrhea, diuretic, and diarrhea diseases (10). The current study confirmed that endophytic bacterium *Streptomyces* sp. strain UICC B-92 isolated from *N. altissima* has the same ability as their host in producing active compounds. It is due to the active compounds produced by the endophytes actually serves to protect their hosts from the various environmental pressures such as plant diseases infection. Further challenges in this study include specific compound determination, mode of action, specificity, and the compound manipulation both physicochemically and genetically to increase yields of desired metabolites.

The phenazine compound synthesized by *Streptomyces* sp. UICC B-92, (4-[(3S,4R,5S)-3,4,5-trihydroxy-6-(hydroxymethyl)tetrahydro-2H-pyran-2-yloxy] phenazine-1-carboxylic acid or 4-O-glucosyl, 1-carboxyl-phenazine), is composed of aromatic compound, phenazine structure, and sugar group. The sugar group in this compound is considered rare due to not many phenazine derivatives synthesized by a member of the genus *Streptomyces* contains sugar structure (18, 19, 27). This phenazine compound (Figs. 3–6) has similarity with glycoside phenazine izuminoside A–C that synthesized by *Streptomyces* sp. IFM 11260 (19), however, a sugar group from *Streptomyces* sp. UICC B-92 belong to is different to that of *Streptomyces* sp. IFM 11260. The sugar group of *Streptomyces* sp. UICC B-92 belong to 4-O-glucosyl and located at the right side of the structure, while the latter belong to rhamnoside and located at the left side (19).

The current study showed that 4-((3S,4R,5S)-3,4,5- trihydroxy-6-(hydroxymethyl)tetrahydro-2H-pyran-2-yloxy) phenazine-1-carboxylic acid or 4-O-glucosyl, 1-carboxyl-phenazine compound exhibited antibacterial activity against two Gram-positive bacteria, *B. cereus* strain ATCC 10876 and *S. aureus* strain ATCC 25923 (Table 2). This compound was produced at the late stage of the *Streptomyces* sp. strain UICC B-92 growth phase. Most of the low molecular weight secondary metabolites produced by microorganisms generally occurs at the late growth phase (13), whereas phenazine production in liquid batch cultures typically occurs after the period of exponential phase (rapid growth), and phenazines are accumulated in the culture at the stationary phase. Phenazine [(C _6_ H _4 _) _2_ N _2_ ] is an organic compound that possess important biological activities such as anti-tumor activity, and a broad-spectrum antimicrobial activity which is important for agricultural and medicine (28, 29). These include streptophenazine A (30), phenazine-1-carboxylic acid (31), antibiotic griseolutein (32), antibiotic lomofungin, endophenazines A–D (16), phenazinolins A–E (7), phenaziterpenes A–B (17). Currently, over 100 different phenazine derivatives have been identified from various sources in nature (33).

A detail antibacterial mechanism of the phenazine compound from *Streptomyces* sp. UICC B-92 is still unknown. However, the results from SEM analysis showed this compound damaged cell walls probably through a kind of lysis mechanism. A morphological change was apparent in the bacterial cells after treatment with the phenazine compound from *Streptomyces* sp. UICC B-92. Occurrence of local rupture or pore formation in the cell membranes is related to leakage of essential intracellular constituents from the cells. This condition causes failure for budding formation, nutrition absorption inability by the cells, and the reduction of the cell walls the metabolism rate and permeability (34), (35). In the end, these conditions cause bacterial cell death.

Many bacterial species produce one or more of the 50 known phenazine compounds, including *Pseudomonas* spp., *Streptomyces* spp., *Nocardia* spp., *Sorangium* spp., *Brevibacterium* spp., *Burkholderia* spp. and *Pantoea agglomerans* (36). Many phenazine-producing microorganisms belong to the bacterial phyla *Actinobacteria* and *Proteobacteria*, and the archaeal phylum *Euryarcheota* (37). The phenazine production among species, isolates, and even repeat cultivations of the same strain are often variable (38). It is probably due to subtle discrepancies in the regulatory networks and sensing mechanisms of the same strain (38). In natural systems, multiple derivatives or an individual phenazines play multiple roles in the bacterial interactions and behaviors (39), for example member of the genus *Streptomyces* (40). In addition, the differences between the gene clusters responsible for the phenazines biosynthesis among different *Streptomyces* species (13) that may lead to the diverse phenazine structural derivatives produced by the bacteria at different levels (39).

Although phenazines are generally isolated from *Pseudomonas, Streptomyces* and several other microbial genera from soil and marine habitats, however, many endophytic *Streptomyces* species associated with various plants are also capable in producing this secondary metabolite compounds (12, 13). This suggests that the endophytic microbial group is one source of potential natural sources in the discovery of new antimicrobial compounds. Strobel and Daisy (3) previously suggested that exploration and conservation of hidden microbial diversity such as microbial endophytes are urgently needed, because they might possess many unknown natural products that are useful for medicines and agrochemical agents. In addition, based on the complexity in a structure, the phenazines from *Streptomyces* are considered more promising than those of *Pseudomonas* (39, 41). Although an investigation on the biosynthesis of 4-((3S,4R,5S)-3,4,5-trihydroxy-6-(hydroxymethyl)tetrahydro-2H-pyran-2-yloxy) phenazine-1-carboxylic acid or 4-O-glucosyl, 1-carboxyl-phenazine compound and determination of genes that responsible for the phenazine biosynthesis in *Streptomyces* sp. strain UICC B-92 is still ongoing, however, the current antibacterial assay showed that this novel compound possesses a potential in the discovery of new agrichemical and medicinal compounds.

In conclusion, this research showed that, bioactive compound of *Streptomyces* sp. strain UICC B-92 is suggested a new compound based on glycoside structure and its position.
